# Editorial: Methods and applications of tumour metabolic imaging in the preclinical and clinical setting

**DOI:** 10.3389/fmed.2024.1536630

**Published:** 2025-01-06

**Authors:** Liangjie Lin, Zhiliang Wei, Jing Wang, Nian Wang

**Affiliations:** ^1^Clinical and Technical Support, Philips Healthcare, Beijing, China; ^2^Russell H. Morgan Department of Radiology and Radiological Science, Johns Hopkins University School of Medicine, Baltimore, MD, United States; ^3^F. M. Kirby Research Center for Functional Brain Imaging, Kennedy Krieger Research Institute, Baltimore, MD, United States; ^4^Department of Radiology, Union Hospital, Tongji Medical College, Huazhong University of Science and Technology, Wuhan, China; ^5^Hubei Province Key Laboratory of Molecular Imaging, Wuhan, China; ^6^Advanced Imaging Research Center, The University of Texas Southwestern Medical Center, Dallas, TX, United States; ^7^Department of Biomedical Engineering, The University of Texas Southwestern Medical Center, Dallas, TX, United States; ^8^Peter O'Donnell Jr. Brain Institute, The University of Texas Southwestern Medical Center, Dallas, TX, United States

**Keywords:** magnetic resonance imaging, metabolic imaging, positron emission tomography, metabolic pathway, cancer metabolism

It is becoming increasingly evident that altered metabolism contributes to tumor progression and has been recognized as a hallmark of cancer. Cancer metabolic heterogeneity may result from genetic diversity, complex metabolic pathways, and altered microenvironmental conditions in tumors. Imaging of tumor metabolism has been remarkably successful in recent years. Numerous studies have demonstrated that malignant tumors can be detected with high sensitivity and specificity by imaging their increased metabolic rates for glucose, amino acids, or lipids (1–3). However, there remains a need for extended studies of the metabolic dependencies of human cancers *in vivo* to understand how metabolism shapes and defines cancer initiation, progression, and response to treatment. Several non-invasive imaging techniques can provide both functional and anatomical information related to tumor metabolism, including positron emission tomography (PET), magnetic resonance imaging (MRI), computed tomography (CT), and optical imaging utilizing bioluminescence. These methods may hold the key to helping improve cancer diagnosis and treatment. In this Research Topic “*Methods and applications of tumor metabolic imaging in the preclinical and clinical setting*”, researchers have demonstrated the potential of different metabolic imaging methods in preclinical and clinical setups for the diagnosis and treatment of a range of tumors.

Neoadjuvant chemotherapy (NAC) has become the standard treatment option for locally advanced breast cancer. Early and accurate prediction of tumor response to NAC is critical for treatment management. Currently, there is still no standard method or imaging biomarker in clinical practice to accurately predict the pathological complete response (pCR) to NAC in breast cancer patients. Amide proton transfer-weighted (APTw) imaging, which detects the exchange between water protons and amide protons in endogenous mobile proteins or polypeptides in tissues, enables indirect detection of changes in protein synthesis and related pathophysiological features in living cells. Zhang et al. hypothesized that APTw imaging may be a potential tool for assessing breast cancer response (especially the early response) to NAC. The results demonstrated that high sensitivity performance can be achieved by APTw for early prediction of MHR status at the end of the first two NAC cycles, which may allow timely regimen refinement before definitive surgical treatment. APTw in combination with tumor diameter and diffusion-weighted imaging may further improve diagnostic accuracy.

The benefits of specifically targeting and boosting resistant hypoxic tumor subvolumes have been promising in clinical trials, but are inconclusive. It is necessary to carry out a comprehensive investigation of the efficacy of boosting hypoxic subvolumes defined by electron paramagnetic resonance oxygen imaging (EPROI). The study by Gertsenshteyn et al. presented data in three preclinical mammalian tumor types to demonstrate that accurate targeting of hypoxic tumor subvolumes with a boost of radiation improves the probability of local tumor control compared to boosting the same integral dose to oxygenated regions of the tumor. This provides additional biological validation of EPROI in targeting enough resistant hypoxic tumor subvolumes to increase the probability of eliminating all clonogens. It may provide a means to reduce the dose to oxygenated regions and deliver a radiation boost to resistant hypoxic regions. Boosting hypoxic subvolumes as defined by EPROI may provide a means to reduce the dose to oxygenated regions and deliver a radiation boost to resistant hypoxic regions. And it may also help reduce the dose to potential organs at risk when tumor control is improved, thus hopefully improving the therapeutic ratio.

Accurate staging of prostate cancer (PCa) is critical for disease management and treatment planning. Prostate-specific membrane antigen (PSMA) is a transmembrane glycoprotein that is overexpressed in prostate cancer cells. PSMA PET/CT, with its excellent sensitivity and specificity, has now become the preferred staging modality for prostate cancer. Compared to other PSMA-targeted tracers, ^18^F-PSMA-1007 is mainly cleared by the liver and bile (reduced urinary radioactive interference), thus allowing for better assessment of lesions in the prostate and pelvic regions. However, significant concentrations of ^18^F-PSMA-1007 may still be observed in the bladder of some patients, which may affect the detection of lesions in the prostate and adjacent areas, especially in patients with clinical suspicion of biochemical recurrence, as long-term androgen deprivation therapy will significantly reduce the visibility of prostate lesions on PSMA PET/CT. Dang et al. proposed to explore the cause of the high uptake of ^18^F-PSMA-1007 in bladder urine by assessing the clinical characteristics and imaging characteristics of PCa patients. The results showed no significant correlation between the high uptake of ^18^F-PSMA-1007 in bladder urine and age, height, weight, Gleason score, metastases, treatment methods, liver and kidney function levels, TPSA levels, ^18^F-PSMA-1007 injected activity, the interval from injection to scan, the physiological distribution of parotid gland, kidney, liver, spleen, intestine, obturator internus, and the pathological distribution of prostate lesions. Therefore, more research is still needed to find out the causes of this problem and to improve the disease assessment in PCa patients.

PET detection of the radioactive glucose analog 2-^18^F-fluoro-2-deoxy-d-glucose (^18^FDG) is now widely used for tumor metabolic imaging. However, ^18^FDG-PET often provides ambiguous results in organs with intrinsically high glucose uptake (such as the brain) and does not provide information on metabolism downstream of glucose uptake. Deuterium metabolic imaging (DMI), which combines deuterium magnetic resonance spectroscopic imaging with oral intake or intravenous infusion of non-radioactive ^2^H-labeled substrates, can non-invasively reveal the glucose metabolism flux by the measurement of downstream deuterated metabolites. A great example is the detection of the Warburg effect in cancer metabolism by measuring the conversion of glucose-6,6-D^2^ to deuterated lactate, glutamine, and glutamate. The review by Wan et al. explored the latest developments and applications of DMI in oncology across various tumor metabolic axes, with emphasis on its potential for clinical translation. DMI offers invaluable insights into tumor biology, treatment responses (notably, the early responses to immunotherapy), and prognostic outcomes. The conclusion is that DMI may evolve into a convenient and efficient imaging technique and promote precision medicine by improving the diagnosis and evaluation of cancer treatments.
